# Characterisation of a Novel Anti-CD52 Antibody with Improved Efficacy and Reduced Immunogenicity

**DOI:** 10.1371/journal.pone.0138123

**Published:** 2015-09-15

**Authors:** Robert G. E. Holgate, Richard Weldon, Timothy D. Jones, Matthew P. Baker

**Affiliations:** Antitope Limited, Babraham Research Campus, Cambridge, United Kingdom; Université Paris Descartes, FRANCE

## Abstract

Anti-CD52 therapy has been shown to be effective in the treatment of a number of B cell malignancies, hematopoietic disorders and autoimmune diseases (including rheumatoid arthritis and multiple sclerosis); however the current standard of treatment, the humanized monoclonal antibody alemtuzumab, is associated with the development of anti-drug antibodies in a high proportion of patients. In order to address this problem, we have identified a novel murine anti-CD52 antibody which has been humanized using a process that avoids the inclusion within the variable domains of non-human germline MHC class II binding peptides and known CD4+ T cell epitopes, thus reducing its potential for immunogenicity in the clinic. The resultant humanized antibody, ANT1034, was shown to have superior binding to CD52 expressing cells than alemtuzumab and was more effective at directing both antibody dependent and complement dependent cell cytotoxicity. Furthermore, when in the presence of a cross-linking antibody, ANT1034 was more effective at directly inducing apoptosis than alemtuzumab. ANT1034 also showed superior activity in a SCID mouse/human CD52 tumour xenograft model where a single 1 mg/Kg dose of ANT1034 led to increased mouse survival compared to a 10 mg/Kg dose of alemtuzumab. Finally, ANT1034 was compared to alemtuzumab in *in vitro* T cell assays in order to evaluate its potential to stimulate proliferation of T cells in peripheral blood mononuclear cells derived from a panel of human donors: whereas alemtuzumab stimulated proliferation in a high proportion of the donor cohort, ANT1034 did not stimulate proliferation in any of the donors. Therefore we have developed a candidate therapeutic humanized antibody, ANT1034, that may have the potential to be more efficacious and less immunogenic than the current standard anti-CD52 therapy.

## Introduction

CD52 is a glycosylphosphatidylinositol (GPI) anchored low molecular weight glycoprotein [1] found in abundance on a variety of normal and malignant lymphoid cells, especially B and T cells, and is expressed at very high density [2]. CD52 is also produced by epithelial cells in the epididymis and duct deferens, and is acquired by sperm during passage through the genital tract [2]. Mature CD52 is a very small glycoprotein with a sequence of only 12 amino acids that is heavily glycosylated at Asn-3 and is linked at its C-terminus to a GPI membrane anchor [2]. The exact biological function of CD52 remains unclear but some evidence suggests that it may be involved in T cell migration and co-stimulation [3][4][5].

To date, the most effective CD52 targeted therapy has been alemtuzumab, a humanized monoclonal antibody genetically engineered by grafting rat complementarity determining regions (CDRs) onto human framework regions fused to human IgG1 [6] that binds to an epitope overlapping the C-terminal part of the CD52 peptide along with part of the GPI anchor [7]. Whilst the mechanism of *in vivo* cell killing is unclear, *in vitro* studies have revealed that upon binding to the cell surface CD52, alemtuzumab induces cell destruction via activation of complement dependent cytotoxicity (CDC) [8] and antibody-dependent cellular cytotoxicity (ADCC); however studies in human CD52 transgenic mice have confirmed the importance of ADCC *in vivo*, but suggest that CDC may not be involved [9]. In addition, intracellular signal transduction by ligation of CD52 leading to caspase-8 dependent and independent apoptosis has also been identified as a potential mechanism of cytolytic action by alemtuzumab [10][11].

Due to its significant cytotoxic effects, alemtuzumab is capable of depleting CD52 positive cells *in vivo* and has demonstrated significant activity against a number of B cell malignancies, particularly in refractory and relapsed chronic lymphocytic leukemia (CLL) for which it was previously marketed under the trade name Campath^®^/Campath-1H, as well as other non-malignant hematopoietic disorders [12][13][14]. This antibody has also been utilized in the treatment of a wide range of other diseases including rheumatoid arthritis [15][16][17] non-Hodgkin’s lymphoma [18][19] and T cell lymphoma [20][21]. Most recently, alemtuzumab has been found to be an effective treatment for relapsing-remitting multiple sclerosis, an indication for which it is now licensed under the trade name Lemtrada™ [22][23].

However, despite its clear successes, alemtuzumab has also been shown to result in substantial toxicity due to attendant immunosuppression associated with its use, and in particular, increased risk of viral and other opportunistic infections [24][25][26]. Furthermore, despite being a humanized antibody, immunogenicity is a significant issue. For example, in a single-dose escalation study of alemtuzumab treatment of rheumatoid arthritis, 63% of patients developed anti-drug antibodies (ADA) with an observed reduction in efficacy [27] and in a study of patients with multiple sclerosis, up to 74% patients developed ADAs [28]. Consequently, in order to improve the clinical utility of anti-CD52 antibody therapy, there is a major need for improved anti-CD52 antibodies which are not associated with significant immunogenicity in patients.

One approach to generating non-immunogenic therapeutic antibodies is through rational design of variable region domains whereby the sequence similarity to human sequence is maximised and the incorporation of CD4+ T cell epitopes is avoided (to create so called ‘Composite Human Antibodies’). The presence of CD4+ T cell epitopes has been shown to be a key intrinsic sequence-related factor that supports the development of anti-drug antibodies in patients [29]. The rational design method used creates a humanized sequence using multiple segments of human variable region sequence from databases of unrelated human antibodies. Variable regions of a reference non-human antibody are modelled to determine antigen binding regions. Sequence segments are then sourced from a database of unrelated human antibody variable regions and screened for the presence of potential CD4+ T cell epitopes using *in silico* MHC class II binding prediction tools and databases of CD4+ T cell epitopes [30], with sequence segments containing potential CD4+ T cell epitopes being discarded. Human variable regions comprising ‘composites’ of human sequence segments (minus CD4+ T cell epitopes) are then constructed and tested for similar binding properties to the reference antibody. This approach combines the advantages of both humanization (by making the V regions more human-like) [31] and deimmunization (by removing potential CD4+ T cell epitopes) [32] and aims to develop antibodies with low clinical immunogenicity.

In this paper, a novel murine anti-CD52 antibody is described which shows improved activity compared with alemtuzumab *in vitro* and in a mouse tumour model and, as a result of humanization using Composite Human Antibody technology, shows reduced immunogenicity compared to alemtuzumab in *ex vivo* CD4+ T cell assays [33].

## Materials and Methods

All reagents were purchased from Sigma (Poole, UK) unless otherwise indicated.

Animal studies were carried out in strict accordance with the recommendations in the Guide for the Care and Use of Laboratory Animals of the National Institutes of Health. Immunizations were performed by QED Biosciences (San Diego, CA) in accordance with QED Bioscience’s IACUC-approved protocols. Tumour animal model studies were performed by Charles River Discovery (Morrisville, NC) in accordance with Charles River’s IACUC-approved protocols. Both studies were reviewed and approved by the appropriate IACUC prior to initiation. In both studies, where necessary, mice were sacrificed by cervical dislocation. CD4+ T cell response studies were performed under National Research Ethics Service Approval No. 07/H0304/114 (Cambridgeshire 1 Research Ethics Committee, Fulbourn, Cambridge), whereby buffy coats were obtained from healthy community donors under written consent.

### Cell lines

REH, Raji, HUT-78 and NS0 cell lines were obtained from ECACC (Salisbury, UK) and 293-c18 cells were obtained from LGC Standards (Teddington, UK). NS0 and 293-c18 cells were maintained in DMEM, high glucose without sodium pyruvate, supplemented with 5% ultra-low IgG FBS and antibiotics. REH and Raji cells were maintained in RPMI1640 supplemented with 10% FBS and antibiotics and HUT-78 cells were maintained in IMDM supplemented with 20% FBS and antibiotics. All growth media and supplements were obtained from Life Technologies (Paisley, UK). Alemtuzumab expressing stable NS0 cell lines were generated by electroporation of NS0 cells [34] with pANT antibody expression vectors containing alemtuzumab heavy and light chain V region sequences together with human IgG1 heavy and kappa light chain constant regions. Secreted antibody was purified from the cell culture supernatants by Protein A chromatography (MabSelect SuRe™, GE Healthcare, Little Chalfont, UK). Mouse IgG1 Fc-alemtuzumab chimeric antibody was made in a similar fashion using alemtuzumab heavy and light chain V region sequences together with murine IgG1 heavy and kappa light chain constant regions. Stable CD52 expressing NS0 cell lines (CD52-NS0) were obtained by electroporation of NS0 cells [34] with full length human CD52 cDNA (GenBank NM_001803.2) (synthesised by GeneArt AG, Regensburg, Germany) subcloned into a mammalian expression vector (downstream of a CMV IE promoter and upstream of a human IgG1 polyA).

REH, Raji and CD52-NS0 cells were dilution cloned, assessed by flow cytometry for CD52 expression and high (designated ^++^) CD52 expressing cell lines identified for all three cell types. HUT78 cells were assessed by flow cytometry for CD52 expression and used without dilution cloning. For flow cytometry analysis of dilution cloned cell lines, 3x10^5^ cells (Raji, REH and CD52-NS0) were incubated with alemtuzumab in flow cytometry (FC) buffer (PBS pH 7.4 containing 1% BSA and 0.05% sodium azide) buffer for 1 hour at 4°C following which, cells were washed 3x with FC buffer and stained with goat anti-human IgG (γ-chain specific), F(ab′)_2_ fragment-phycoerythrin antibody (cat. no. P8047) diluted 1 in 100 in FC buffer. Following incubation for 1 hour at 4°C, cells were washed 3x with FC buffer and finally resuspended in the same buffer. Flow cytometry was performed using a FACSCalibur (Becton Dickinson, Oxford, UK) with instrument settings determined by analysis of relevant isotype control antibodies.

### Immunisations and hybridoma selection

CD52 peptide (GQNDTSQTSSPSC) was synthesised and conjugated via the peptide C-terminal cysteine to either KLH or BSA (Mimotopes, Melbourne, Australia). Immunisations were performed by QED Bioscience (San Diego, CA). Female Balb/c mice were primary immunised by intraperitoneal (i.p.) injection with CD52 peptide-KLH conjugate in Complete Freund’s Adjuvant. Mice were subsequently boosted by i.p. injection of HUT-78 cells followed by a three further boosts with CD52-NS0^++^ cells. Mice showing the highest antibody titres were sacrificed by cervical dislocation, and the cells from each entire spleen were pooled and fused to F0 myeloma cells by PEG-mediated fusion. The resulting fusions were seeded into 96-well plates and cultured at 37°C in 5% CO_2_ for two weeks in the presence of hypoxanthine, aminopterin and thymidine. Hybridomas were tested for CD52-specific antibody by CD52 peptide ELISA, CD52-NS0 cell-based ELISA and by flow cytometry, and positive wells selected. Expanded hybridoma cell culture supernatants were harvested, adjusted to pH 7.4 using 10x PBS (Invitrogen, Paisley, UK) and purified using Protein A chromatography (MabSelect SuRe™, GE Healthcare, Little Chalfont, UK).

### CD52 assays

For the CD52 peptide ELISA, 96-well Maxisorp plates (Nunc-Immuno™, Greiner Bio-one, Stonehouse, UK) were coated overnight at 4°C with either CD52 peptide-KLH, CD52 peptide-BSA, KLH only or BSA only in PBS pH 7.4. Plates were washed and blocked with PBS containing 2% BSA. Hybridoma supernatants or purified antibodies were diluted in PBS/2% BSA and added to each plate followed by incubation for 1 hour at room temperature. Plates were washed with PBS/Tween®20 (0.05% v/v) and incubated for 1 hour with goat anti-mouse IgG (Fab-specific)-HRP (cat. no. A9917) diluted 1 in 500 in PBS/2% BSA. Plates were washed with PBS/Tween^®^20 following which SigmaFast OPD substrate was added and incubated at room temperature in the dark to allow colour to develop. The reaction was stopped by adding 3M HCl and plates were read at 490 nm using a Dynex MRXII plate reader (Dynex Technologies, Worthing, UK). Peptide competition assays were similarly performed using a biotinylated (BiotinTag™ micro biotinylation kit) reference antibody that was mixed at a fixed concentration with titrations of test antibody samples and binding was detected with Streptavidin-HRP (cat. no. S5512) diluted 1 in 1000 in PBS/2% BSA and TMB substrate (Invitrogen, Paisley, UK). The reaction was stopped by adding 3M HCl and plates were read at 450 nm using a Dynex MRXII plate reader.

For the CD52 cell-based ELISA, CD52-NS0^++^ cells or untransfected NS0 cells were plated in a V-bottom 96 well plate (Cellstar®, Greiner Bio-one, Stonehouse, UK) and incubated with hybridoma samples diluted 1 in 2 in FC buffer. After incubation at room temperature for 1 hour, the cells were washed 3x by centrifuging the plates and resuspending the cells in FC buffer. After the final wash, cells were resuspended in FC buffer containing goat anti-mouse IgG (Fab specific)-HRP (cat. no. A9917) diluted 1 in 500. After incubation for 1 hour at room temperature, cells were washed by centrifugation, resuspended in PBS pH 7.4 and transferred to an ELISA plate. TMB substrate (Invitrogen, Paisley, UK) was added and incubated at room temperature in the dark to allow the colour to develop. The reaction was stopped by adding 3M HCl and plates were read at 450 nm using Dynex MRXII plate reader.

For flow cytometry binding analysis of antibodies, 3x10^5^ cells (untransfected NS0, HUT78, Raji^++^, REH^++^ or CD52-NS0^++^) were stained either using a 1 in 2 dilution of hybridoma supernatant in FC buffer together with sheep anti-mouse IgG (whole molecule) F(ab′)_2_ fragment-PE antibody (cat no. P8547) diluted 1 in 100 in FC buffer, or using varying concentrations of chimeric or humanized samples together with goat anti-human IgG (γ-chain specific) F(ab′)_2_ fragment-PE antibody (cat. no. P8047) diluted 1 in 100 in FC buffer. Following incubation for 1 hour at 4°C, cells were washed 3x with FC buffer and finally resuspended in the same buffer. Flow cytometry was performed using a FACSCalibur with instrument settings determined by analysis of relevant isotype control antibodies. Flow cytometry competition assays were similarly performed using a mouse IgG1 Fc-alemtuzumab chimeric antibody that was mixed at a fixed concentration with titrations of test antibody samples and binding was detected with sheep anti-mouse IgG (whole molecule) F(ab′)_2_ fragment-PE antibody as above. Flow cytometry was performed using a FACSCalibur with instrument settings determined by analysis of relevant isotype control antibodies.

### Variable region gene sequencing and generation of chimeric antibody

Total hybridoma RNA was extracted using an Ambion RNAqueous-4PCR Kit (Life Technologies, Paisley, UK) and cDNA synthesised using an ImProm-II™ Reverse Transcription System (Promega, Southampton, UK). Murine immunoglobulin heavy and kappa light chain variable (V) region fragments were amplified by RT-PCR using degenerate mouse leader sequence primers and unique constant domain primers [35]. Amplified fragments were cloned into pGEM^®^-T Easy (Promega, Southampton, UK) and sequenced (Source BioScience, Cambridge, UK). Selected heavy and light chain V region sequences were PCR amplified and subcloned into pANT antibody expression vectors containing human genomic IgG1 heavy and kappa light chain constant regions downstream of a CMV IE promoter (pANT17 and pANT13 respectively). The heavy and light chain expression constructs were either transiently co-transfected into 293-c18 cells using calcium phosphate precipitation [36] or electroporated into NS0 cells [34] to create stable cell lines. Secreted antibody was purified from the cell culture supernatants by Protein A chromatography (MabSelect SuRe™, GE Healthcare, Little Chalfont, UK).

### Antibody humanization/deimmunisation

To create humanized variants using Composite Human Antibody™ technology, structural models of the mouse V regions were produced using Swiss-PDB Viewer (http://www.expasy.org/spdbv/) [37] and analysed in order to identify amino acids that were likely to be important for the CD52 binding properties of the antibody (‘constraining residues’). A database of human V region sequences was used to identify segments (typically 3–20 amino acids long) of human V region sequences containing each of the constraining residues. Typically two or more alternative V region sequence segments were used to provide each constraining residue resulting in a large range of possible sequences of humanized anti-CD52 V region sequences. Segments were then analysed for the prediction of non-germline MHC class II peptide binding by iTope *in silico* analysis [33] and also for known CD4+ T cell epitopes using databases including TCED™ [30]. V region sequences with predicted non-germline MHC class II binding peptides, or with significant hits against T cell epitope databases were discarded resulting in a reduced set of V region sequences, selected combinations of which were then combined to produce humanized/deimmunized (hereinafter termed ‘humanized’) heavy and light chain variable region amino acid sequences. Humanized variant V regions were synthesised and subcloned into the expression vectors pANT17 and pANT13 as described above. All combinations of humanized VH and Vκ chains were transiently transfected into 293-c18 cells [36] and also transfected into NS0 cells for stable cell lines [34]. Antibody was purified by protein A chromatography (MabSelect SuRe™, GE Healthcare, Little Chalfont, UK) from the culture supernatants.

### Antibody-dependent cellular cytotoxicity (ADCC)

Assays were performed essentially as described previously [38], except that target cells were labelled with Calcein-AM (Sigma, Poole, UK). Briefly, the target cell line (either REH^++^ or Raji^++^ cells) were harvested and preloaded with 25 μM (final concentration) Calcein-AM. After incubation with Calcein-AM for 1 hour at 37°C, cells were washed in media to remove unincorporated dye and resuspended in growth medium in the presence or absence of test antibodies. Following incubation for 1 hour at room temperature, cells were washed again and plated at 1x10^4^ cells/well in the presence of 5x10^5^ freshly isolated peripheral blood mononuclear cells (PBMC) from normal blood donors (50:1 ratio of effector to target cells). After a 4 hour incubation at 37°C, Triton™ X-100 (Sigma, Poole, UK) was added to the control wells containing cells (effector and/or target cells) to establish the maximum lysis control. Supernatants were transferred into a flat-bottomed 96-well plate and the fluorescence was read at 520 nm in a Fluostar Optima fluorimeter (BMG Labtech, Aylesbury, UK).

### Complement-dependent cytotoxicity (CDC)

Target cells (either REH^++^ or Raji^++^) were plated out at 5x10^4^ cells per well. Test antibodies were added at the indicated final concentrations together with either active or heat inactivated (60°C for 30 minutes) human serum **(**Pathway Diagnostics, Dorking, UK) (25% final serum concentration). Plates were incubated for 3 hours at 37°C, following which PrestoBlue® cell viability reagent (Invitrogen, Paisley, UK) was added. Triton® X-100 was added to the control cells-only wells to establish the maximum lysis control. After incubation for 1 hour at 37°C, fluorescence was measured at 590 nm in a Fluostar Optima fluorimeter (BMG Labtech, Aylesbury, UK).

### Direct cell killing

Direct cytotoxic effects of anti-human CD52 antibodies were assessed via flow cytometry using Annexin V and Propidium Iodide co-staining as markers of apoptosis and necrosis respectively. REH^++^ cells were plated in the presence of 100 μg/ml test antibodies either with or without 100 μg/ml goat anti-human IgG (γ-chain specific) F(ab’)_2_ crosslinking antibody (Stratech Scientific, Newmarket, UK, cat. no. 109-006-008). Cells were incubated for 72 hours before washing in PBS/2%BSA followed by co-staining with Annexin V and Propidium Iodide according to the manufacturer’s recommended protocol (Invitrogen, Paisley, UK). Scatterplots were generated using flow cytometric analysis and divided into three regions for quantitation of live cells (unstained), apoptotic cells (FL1, Annexin V positive) and necrotic cells (FL1, Annexin V positive and FL3, Propidium Iodide positive).

### Tumour animal model

Animal studies were performed by Charles River (Morrisville, NC) using a method described previously [39]. Female Fox Chase SCID mice (8 females per group) were injected with 1x10^6^ Raji^++^ cells via a bolus tail-vein injection. Anti-human CD52 antibodies or an isotype matched control antibody were administered intraperitoneally (i.p.) once daily on alternate days for seven doses, starting three days after tumour cell injection. Animals were monitored daily for signs of tumour progression. Animals showing advanced tumour progression, as manifested by hind limb paralysis, ocular proptosis or moribundity, were humanely euthanized by cervical dislocation. Efficacy was determined by comparing the median times to endpoint (either death, or euthanasia for advanced tumour progression). Over the duration of the study, 2 mice from the alemtuzumab (10 mg/Kg) group, 1 mouse from the ANT1034 (1 mg/Kg) group and 2 mice from the ANT1034 (10 mg/Kg) group died between monitoring checks and before euthanasia could be administered.

### Analysis of CD4+ T cell responses

PBMC from healthy community donor buffy coats were isolated from buffy coats by Lymphoprep (Axis-Shield, Dundee, UK) density centrifugation and CD8+ T cells were depleted using CD8+ RosetteSep™ (StemCell Technologies, Cambridge, UK). Donors were characterized by identifying HLA-DR haplotypes using an HLA SSP-PCR based tissue-typing kit (Biotest, Shirley, UK) and 50 different donor PBMCs were selected to provide a distribution with frequencies of HLA-DR and HLA-DQ allotypes similar to the overall world population.

To prepare monocyte derived dendritic cells (DC), CD14+ cells were isolated from PBMC using CD14 Microbeads and LS columns (Miltenyi Biotech, Bisley, UK). The monocytes were resuspended in DC culture medium (AIM-V^®^ medium; Life Technologies, Paisley, UK) supplemented with 1000 IU/ml IL-4 and GM-CSF (Peprotech, London, UK) and allowed to differentiate into semi-mature DC. Semi-mature DC were incubated with test samples for 24 hours after which excess sample was removed by washing, and the cells were resuspended in DC culture media supplemented with 50 ng/ml TNF-α (Peprotech, London, UK) and incubated for 8 days. DC were then γ-irradiated (4000 rads) and resuspended in AIM-V^®^ media. Additionally, on day 8, fresh CD4+ T cells were prepared: PBMC were revived in AIM-V^®^ culture medium and CD4+ cells isolated by negative selection using a CD4+ T cell isolation kit and LS columns (Miltenyi Biotech, Bisley, UK).

For T cell proliferation assays, 1x10^5^ autologous fresh CD4+ T cells were added to 1x10^4^ irradiated DC cells (pre-loaded with test sample) in 96 well U-bottomed plates in sextuplicate cultures. After an additional 7 days, assay plates were pulsed with 1 μCi [3H] (Perkin Elmer, Seer Green, UK) per well in AIM-V^®^ for 6 hours before harvesting onto filter mats (Perkin Elmer, Seer Green, UK)) using a TomTec Mach III cell harvester. Counts per minute (cpm) for each well were determined by Meltilex™ scintillation counting on a 1450 Microbeta Wallac Trilux Liquid Scintillation Counter (Perkin Elmer, Seer Green, UK) in paralux, low background counting. Counts per minute for each antibody sample were normalised to the media only control. Results were expressed as a Stimulation Index (SI) defined as the ratio of cpm for the test antibody against a medium-only control using an emperical threshold of SI equal to or greater than 1.9 (SI≥1.90) for positive T cell responses. The threshold of SI ≥ 1.9 has been previously established whereby test samples inducing responses above this threshold are deemed positive. Extensive assay development and previous studies have shown that this is the minimum signal-to-noise threshold allowing maximum sensitivity without detecting large numbers of false positive responses or omitting subtle immunogenic events. For proliferation (n = 6), positive responses were defined by statistical and empirical thresholds as follows:

1. Significance (*p* < 0.05) of the response by comparing cpm of test wells against medium control wells using unpaired two sample Student’s t-test.
2. Stimulation index greater than or equal to 1.9 (SI ≥ 1.90), where SI = mean of test wells (cpm) / baseline (cpm). Data presented in this way is indicated as SI ≥ 1.90, *p* < 0.05.

## Results

### Cell line analysis

**Fig 1:**
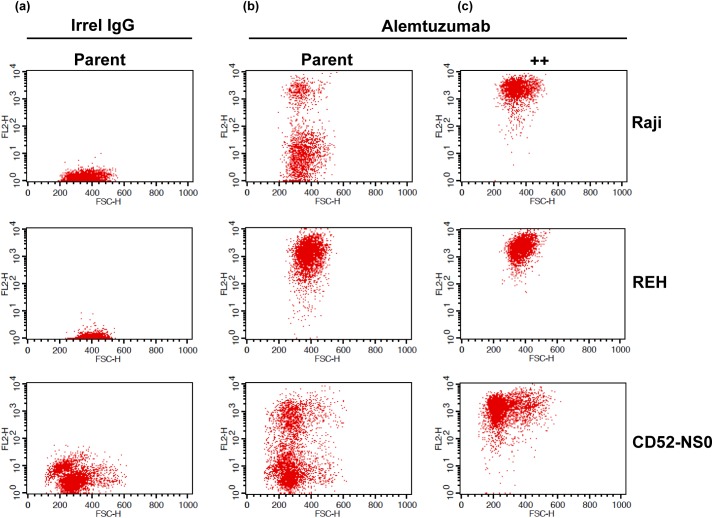
Selection of high CD52-expressing cell lines. Raji, REH and CD52-NS0 cells were assessed by flow cytometry for CD52 expression and high CD52 expressing (^++^) clones were identified for the three cell lines. Cells (either parent or ^++^ lines) were stained with irrelevant human IgG (a) or with alemtuzumab (b) and (c).

Many *in vitro* maintained lymphoid-derived tumour cells have been shown to express CD52, however the level of expression can vary significantly and the stability of the expression is unpredictable. To overcome this heterogeneity, CD52 expressing cell lines were analysed for levels of CD52 expression and CD52 high expressing (designated ^++^) clones were identified for Raji, REH and CD52-NS0 cell lines (Fig 1).

### Hybridoma generation and selection

**Fig 2:**
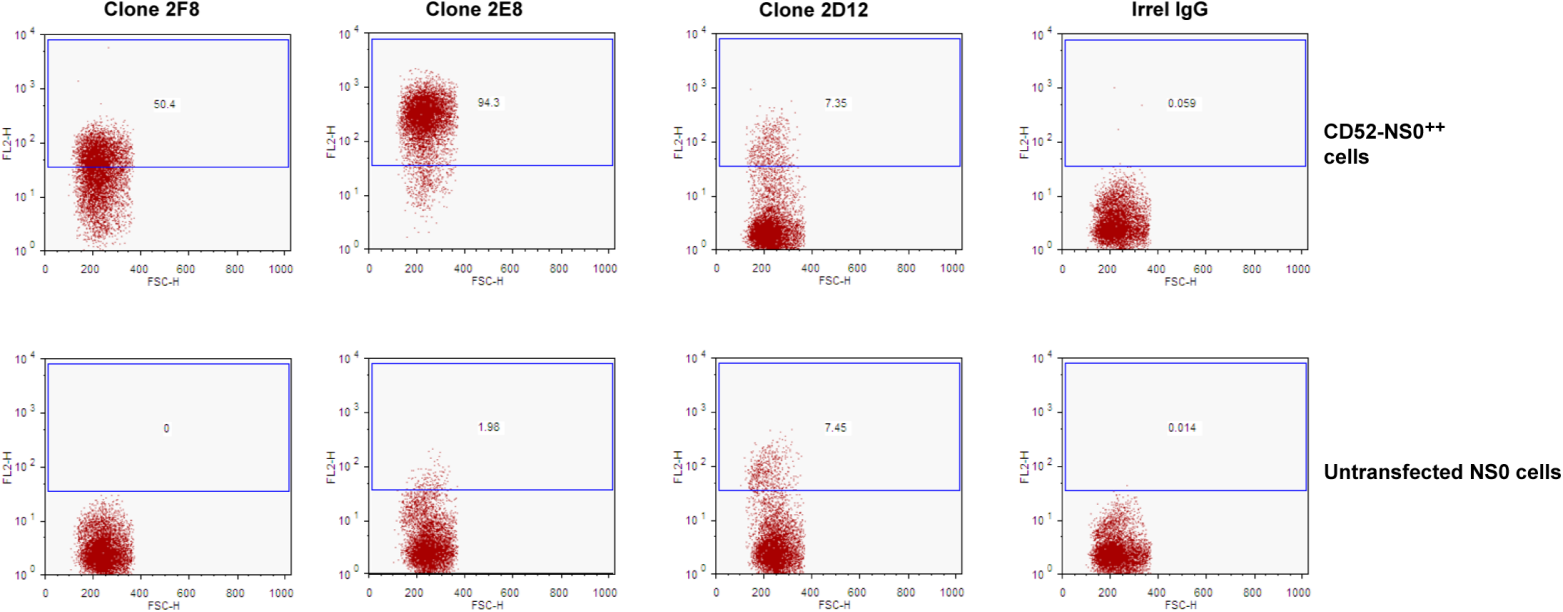
Flow cytometry analysis of hybridomas or irrelevant mouse IgG. Selected hybridoma supernatants or media spiked with irrelevant mouse IgG were assessed for binding to either untransfected or NS0-CD52^++^ cells by flow cytometry to identify CD52 binding antibodies.

Mice were immunised using a combination of CD52 peptide (conjugated to KLH), HUT-78 cells and murine NS0 cells expressing full length human CD52 (including the N-terminal signal peptide and the C-terminal displaced GPI-anchor signal peptide allowing for the production of mature GPI-anchored surface peptide). The mouse with the highest anti-peptide ELISA titres was sacrificed and derived spleen cells immortalised by fusion with myeloma cells. Hybridomas were selected for supernatant binding activity to CD52 using both a peptide ELISA (CD52-specific hybridomas were those that bound to CD52 peptide-KLH and CD52 peptide-BSA but not to either KLH or BSA alone) and a cell based ELISA (CD52-specific hybridomas were those that specifically bound to CD52-NS0 cells but not to untransfected NS0 cells). Several hybridomas were identified that bound CD52 either as peptide or on cells however, one hybridoma in particular, clone 2E8, was identified as secreting antibody that strongly bound CD52 in both assay formats. Selected assay positive hybridomas were analysed further by flow cytometry on CD52-NS0 and untransfected NS0 cells (Fig 2).

### Generation and characterisation of chimeric 2E8 antibody

**Fig 3:**
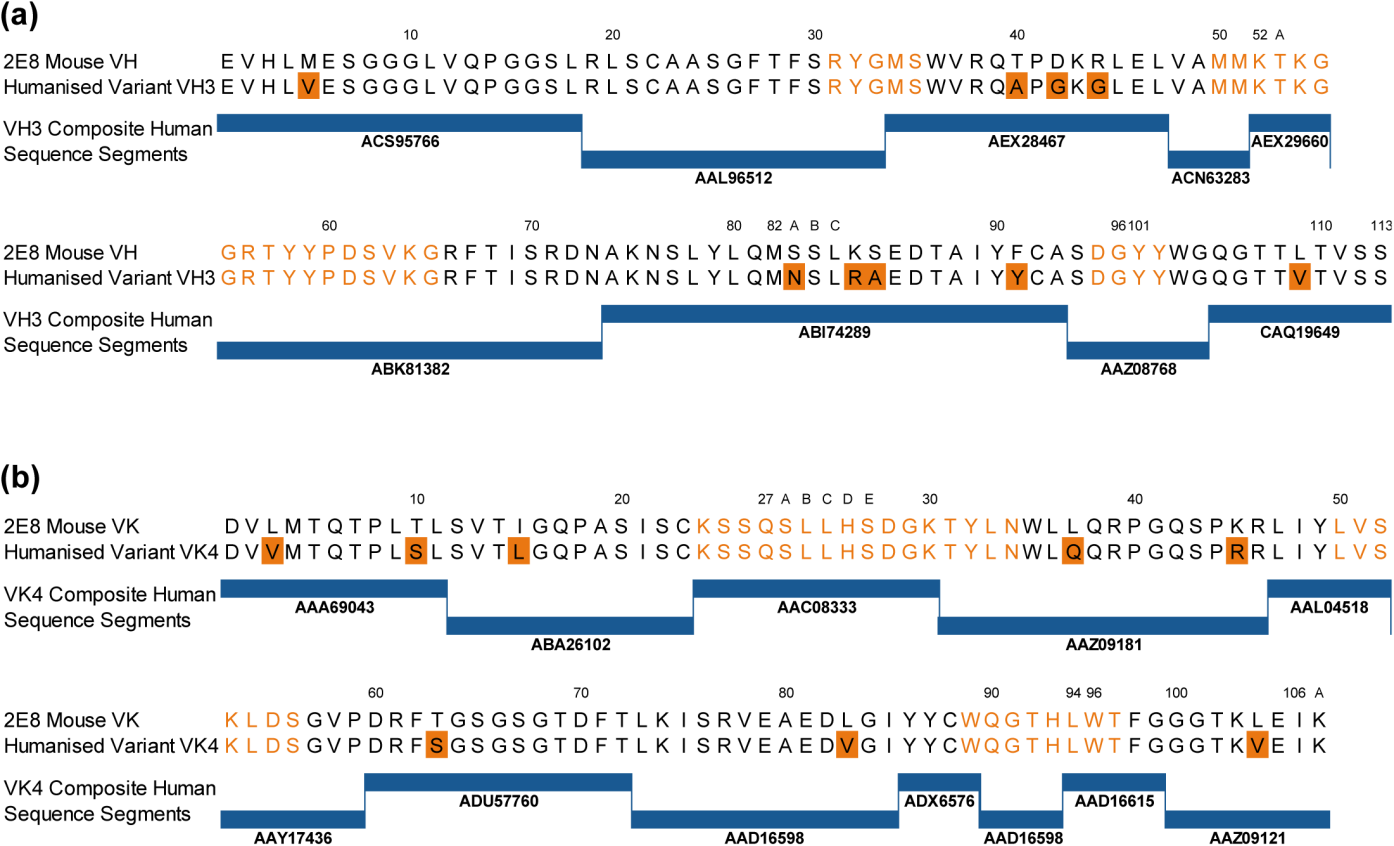
Amino acid sequence of murine anti-CD52 antibody 2E8 and lead humanized variant. Amino acid sequences of the murine antibody 2E8 and lead humanized variant (ANT1034 –composed of VH3 and VK4 variable domains). Sourced human sequence segments together with their GenBank accession numbers are indicated below the amino acid sequences. (a) Heavy chain variable domain; (b) Light chain variable domain. Differences between the murine and humanized sequence are shaded. CDRs (shown in orange text) and numbering are as described by Kabat [41]. Sequences are available from GenBank: 2E8_VH—KP877884; 2E8_VK—KP877885; ANT1034_VH—KP877886; ANT1034_VK—KP877887.

2E8 antibody variable domain genes were amplified, cloned and sequenced (Fig 3) and analysis of the derived sequences showed that VH CDR3 was considerably shorter than the average mouse VH CDR3 (4 amino acids compared with an average of 11.5±1.9 amino acids [40]). Comparison with germline sequences revealed that the VH region had undergone significant somatic hypermutation particularly within VH CDR2, suggesting that this CDR may play a critical role in binding to the antigen.

**Fig 4:**
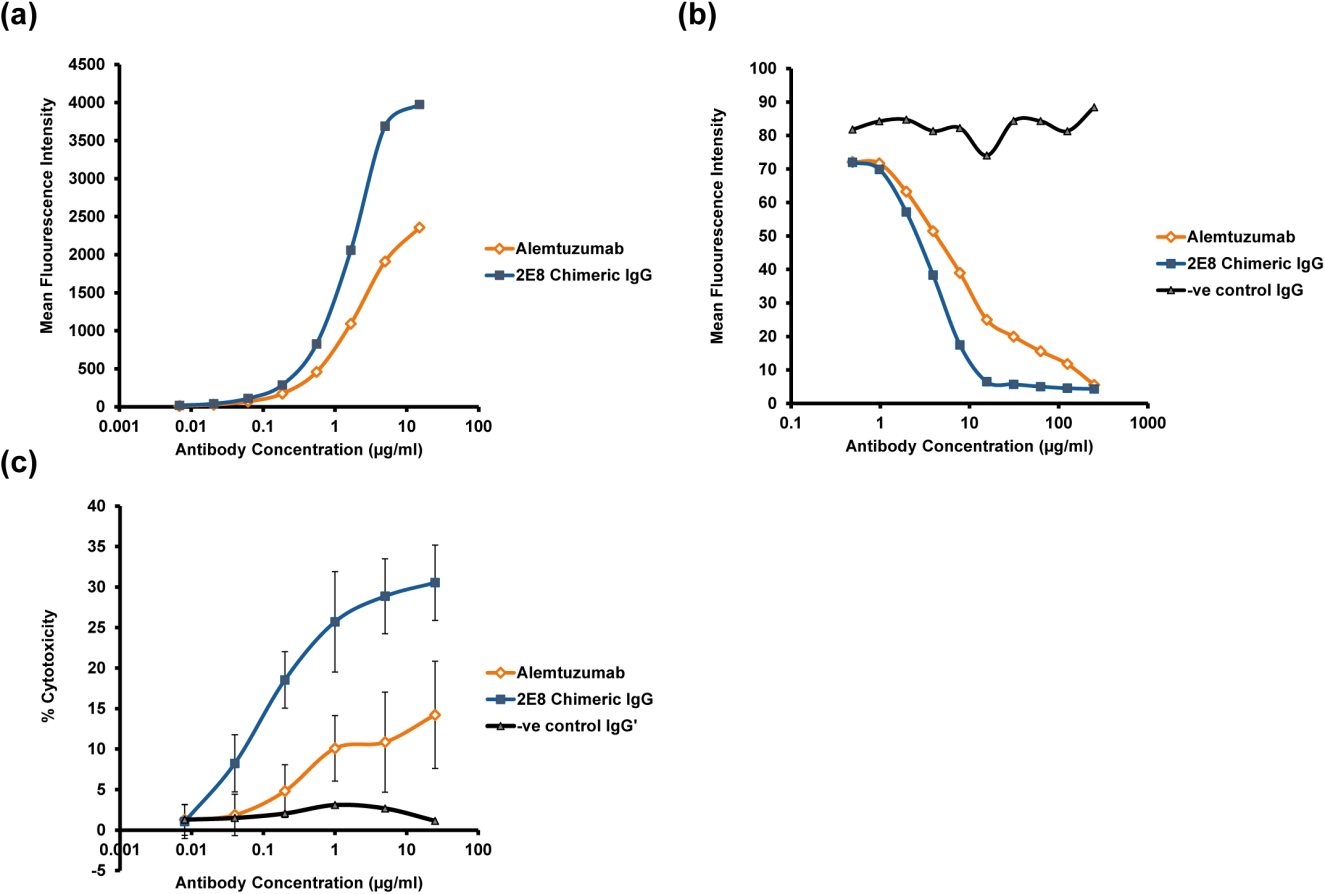
Characterisation of anti-CD52 antibodies alemtuzumab and chimeric 2E8 IgG. Antibodies were characterised by: (a) Direct binding of antibodies to HUT78 cells using flow cytometry; (b) Competition against alemtuzumab (with murine constant regions) for binding to CD52 on the surface of HUT78 cells using flow cytometry; (c) ADCC using REH^++^ target cells (average of 4 PBMC donors for effector cells).

The heavy and light chain V region sequences of the 2E8 murine monoclonal antibody were subcloned into expression vectors containing human IgG1 heavy and kappa light chain constant regions. The resultant chimeric antibody was expressed in, and purified from 293-c18 cells. The binding of the chimeric antibody was compared to alemtuzumab (produced in the same way) in both a direct flow cytometry binding assay and competition assay (Fig 4). The chimeric 2E8 antibody exhibited greater direct binding to HUT78 cells (Fig 4A) and an improved ability to compete with alemtuzumab for binding to CD52 (Fig 4B).

In addition to being able to bind cell-surface CD52, an important mechanism of action for alemtuzumab is through its ability to mediate ADCC. Therefore, the chimeric 2E8 and alemtuzumab antibodies were compared for their ability to induce cytotoxicity using PBMC effector cells and REH^++^ target cells and it was found that chimeric 2E8 showed significantly enhanced ADCC compared to alemtuzumab (Fig 4C). Taken together, the improved ability to bind surface CD52 and functional data suggested that this clone showed significant potential as a candidate therapeutic antibody.

### Humanization of 2E8

Humanized 2E8 antibody V region sequences were designed to be devoid of non-germline MHC class II peptide binding peptides and known T cell epitopes thereby reducing their potential for immunogenicity. Selected sequence segments were assembled into complete V region sequences resulting in a series of five humanized heavy chains and four humanized light chain sequences (VH1 to VH5 and Vκ1 to Vκ4 respectively) which were synthesised and cloned into human IgG1 / kappa expression vectors. The lead VH and Vκ variant amino acid sequences together with the human antibodies from which sequence segments were sourced are shown in Fig 3. All combinations of heavy and light chains were expressed transiently in 293-c18 cells and stable cell lines were prepared in NS0 cells. All antibodies were purified by Protein A affinity chromatography.

### Characterisation of humanized antibodies

**Fig 5:**
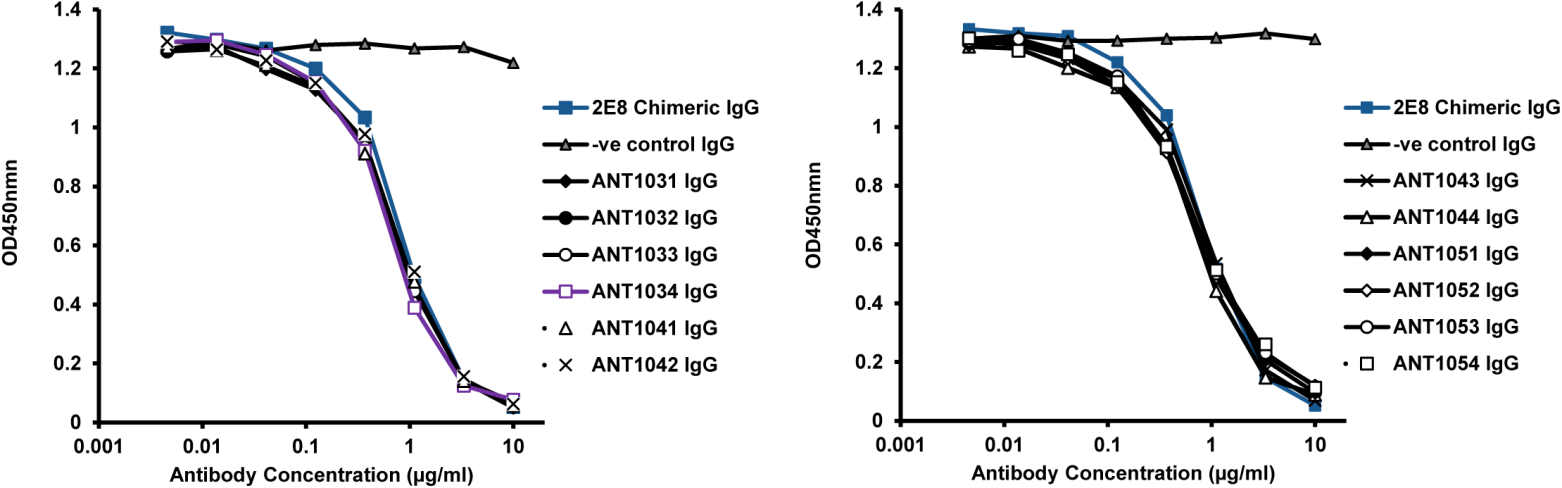
Peptide competition ELISA of selected humanized variants. Titrations of humanized variants were competed against a fixed concentration (35 ng/ml) of biotinylated murine 2E8 for binding to CD52 peptide that was coated directly on an ELISA plate. Binding was detected with streptavidin-HRP and TMB substrate. Antibodies are named in the format ANT10X_1_X_2_ where X_1_ refers to the VH variant (VH1 to VH5) and X_2_ refers to the Vκ variant (Vκ1 to Vκ4).

**Fig 6:**
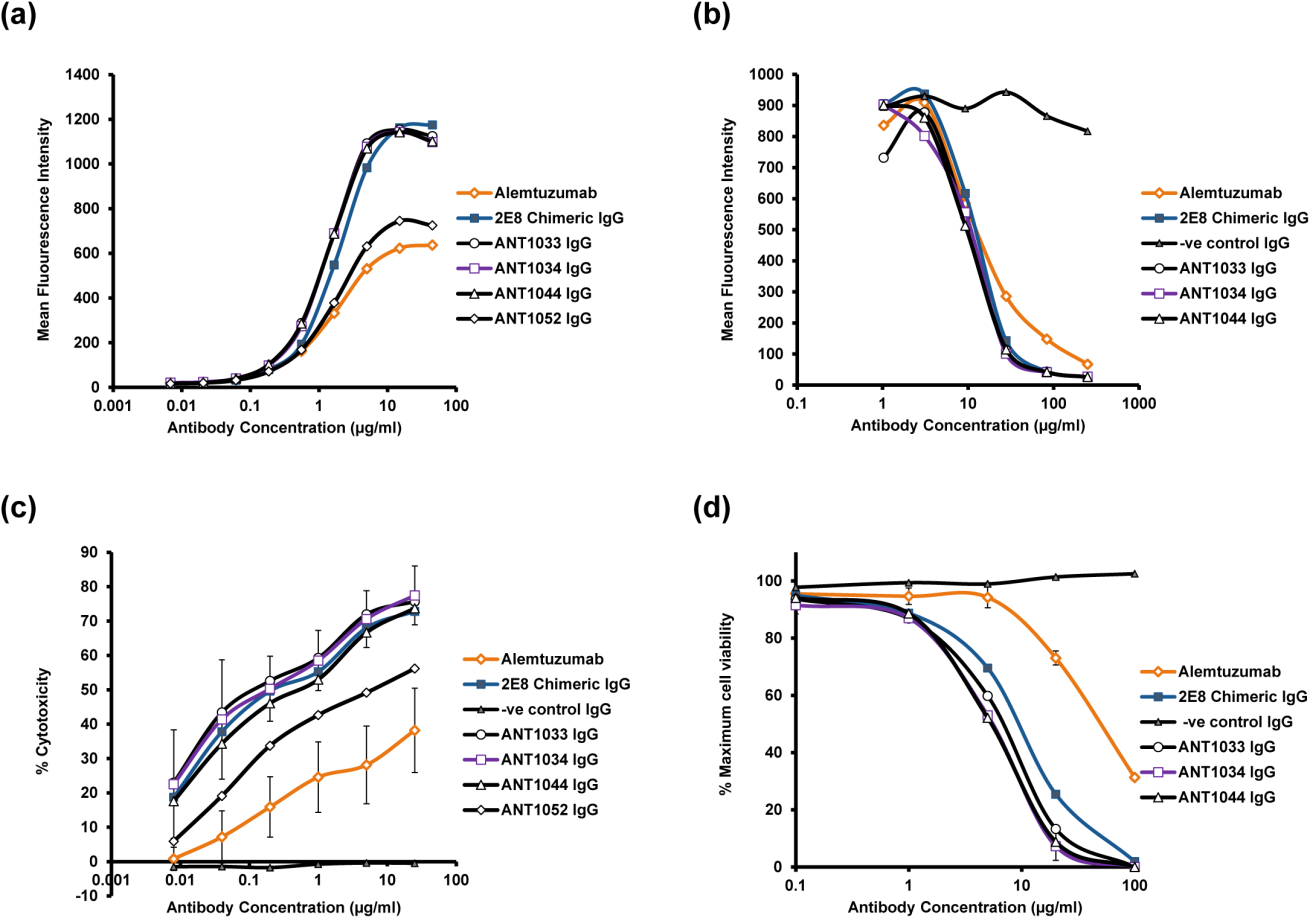
Characterisation of anti-CD52 humanized antibodies, alemtuzumab and 2E8 chimeric IgG. Selected humanized antibodies were characterised by: (a) Direct binding of antibodies to REH^++^ cells using flow cytometry; (b) Competition binding against alemtuzumab (with murine constant regions) for binding to CD52 on the surface of REH^++^ cells using flow cytometry; (c) ADCC using REH^++^ target cells (average of 4 PBMC donors) with PBMC effector cells at a target:effector ratio of 50:1, and; (d) CDC using REH^++^ target cells and normal human serum as a source of complement. For clarity, error bars are included in (c) and (d) for alemtuzumab and the lead humanized antibody, ANT1034, only.

As the 2E8 hybridoma showed the distinctive feature of being able to bind to CD52 peptide (unlike alemtuzumab), the binding of all of the humanized variants to CD52 peptide was assessed in a competition ELISA against the parent 2E8 chimeric antibody. All lead humanized 2E8 variants displayed competitive binding profiles similar to the parent chimeric antibody (Fig 5); to discriminate further between the humanized variants and to compare directly with alemtuzumab, the antibodies were subsequently tested for binding to REH^++^ cells by flow cytometry (either direct binding or in competition with alemtuzumab) and for effector function in ADCC and CDC assays (Fig 6). The chimeric antibody and the majority of humanized variants exhibited an improved binding profile by flow cytometry compared to alemtuzumab (Fig 6A) and also competed effectively with alemtuzumab for binding to CD52 (Fig 6B). The humanized variants also exhibited improved ADCC (in excess of 100 fold) using REH^++^ target cells and PBMC effector cells at a target:effector cell ratio of 50:1 (Fig 6C). Similarly, when using the REH^++^ cell line for assessment of CDC, the humanized variants showed an approximate 10-fold increase in cytotoxicity when compared to alemtuzumab (Fig 6D). Heat inactivating the human serum prior to use in the CDC assay resulted in no significant lysis, thus indicating the requirement of active complement for cell killing.

Interestingly, the cell-based assays were able to discriminate between variants where the peptide competition ELISA could not. In particular, it was observed that the humanized variants containing the VH5 heavy chain, which differs from all other variants by the presence of a tryptophan at position 47 (Kabat) in place of a leucine, showed reduced binding by flow cytometry (Fig 6A) and reduced activity in the ADCC assay (Fig 6C) compared to the other humanized variants and chimeric IgG; however the VH5 variants were still improved compared to alemtuzumab. Based on this reduction in activity, the VH5-containing variants were not selected as potential lead antibodies.

### Direct killing using anti CD52 antibodies

**Fig 7:**
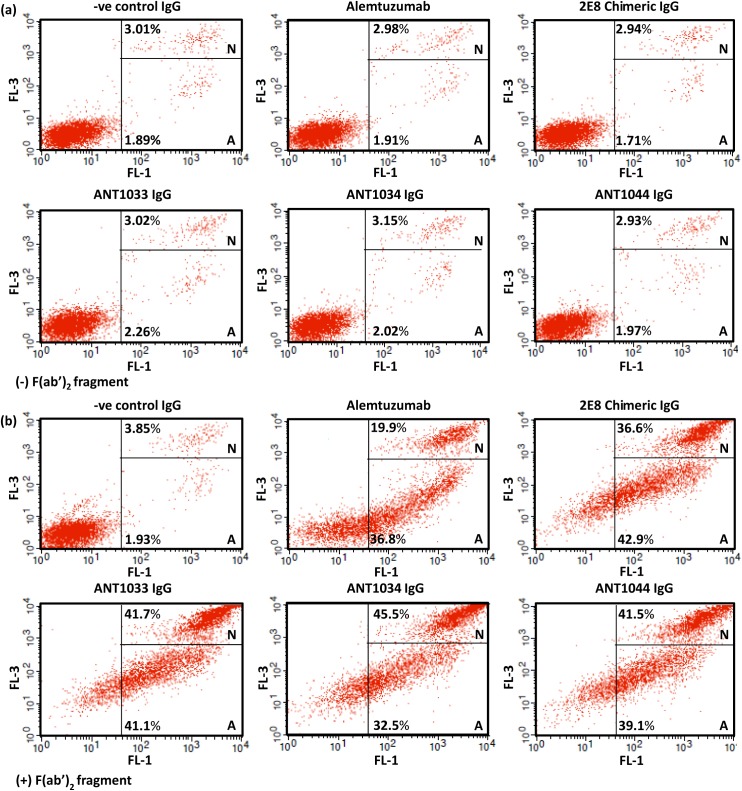
Flow cytometry analysis of REH^++^ cells incubated with selected humanized antibodies and alemtuzumab. Cells were stained with Annexin V as a marker of apoptosis (gate = A) and Propidium Iodide as a marker of necrosis (gate = N). (a) Analysis in the absence of a cross-linking antibody; (b) Analysis with a cross-linking antibody. The percentage of cells in each gate is indicated.

It has been reported previously [10][11] that alemtuzumab significantly enhances apoptosis in chronic lymphocytic leukemia cells *in vitro*, especially in combination with a cross-linking anti-Fc-antibody, with this effect being mediated by a caspase-dependent pathway. In order to investigate whether the novel CD52 antibody could exert a similar property, the ability of selected humanized anti-human CD52 antibodies to mediate direct cytotoxic effects was assessed using flow cytometry with Annexin V and Propidium Iodide co-staining as markers of apoptosis and necrosis respectively (Fig 7). In the absence of cross-linking antibody, very little apoptosis or necrosis was detectable with no significant differences observed between alemtuzumab, 2E8 chimeric antibody and the humanized variants (Fig 7A). However, in the presence of cross-linking antibody, the proportion of cells undergoing apoptosis and necrosis increased significantly, with the percentage of necrotic cells in the humanized antibody treated samples being in excess of 40% compared to ~20% for the alemtuzumab treated sample (Fig 7B). Furthermore, fewer unstained cells were present in the humanized antibody treated samples indicating a more rapid progression through apoptosis and necrosis compared to the alemtuzumab treated sample. Considering all the *in vitro* data together, humanized antibody ANT1034 was selected for further study in a SCID mouse tumour model.

### *In vivo* studies using anti CD52 antibodies

**Fig 8:**
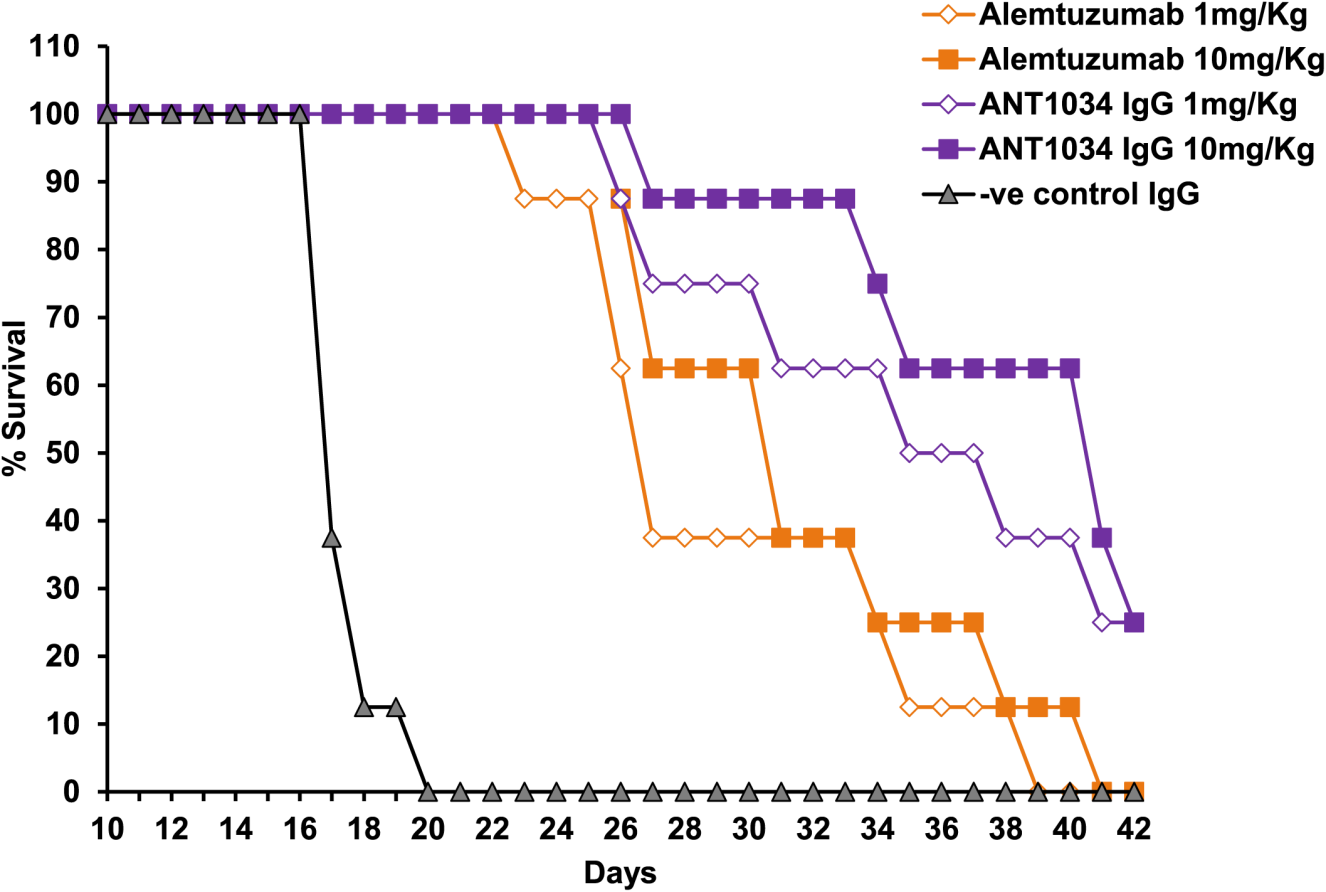
Kaplan-Meier survival curves for the *in vivo* assessment of ANT1034 and alemtuzumab. Antibodies were assessed in the Raji human Burkitt lymphoma SCID mouse model. Antibodies were administered on alternate days for seven doses starting 3 days post-injection of tumour cells. Efficacy was determined by comparing the median times to endpoint (either death, or euthanasia for advanced tumour progression).

ANT1034 and alemtuzumab were compared in a Raji human Burkitt lymphoma SCID tumour xenograft mouse model [39] to measure the effect on tumour inhibition. Animals were treated with anti-human CD52 antibodies on alternate days for seven doses starting 3 days after injection of tumour cells. The results demonstrated an improved survival rate for alemtuzumab **(** Fig 8 **)** (1 and 10 mg/Kg doses shown) compared to the isotype matched IgG1 control antibody with ANT1034 eliciting further enhancements in survival, even at the lower dose. Therefore the improved performance of ANT1034 in comparison to alemtuzumab was observed in both *in vitro* and *in vivo* models.

### Immunogenicity of anti-CD52 antibodies

**Fig 9:**
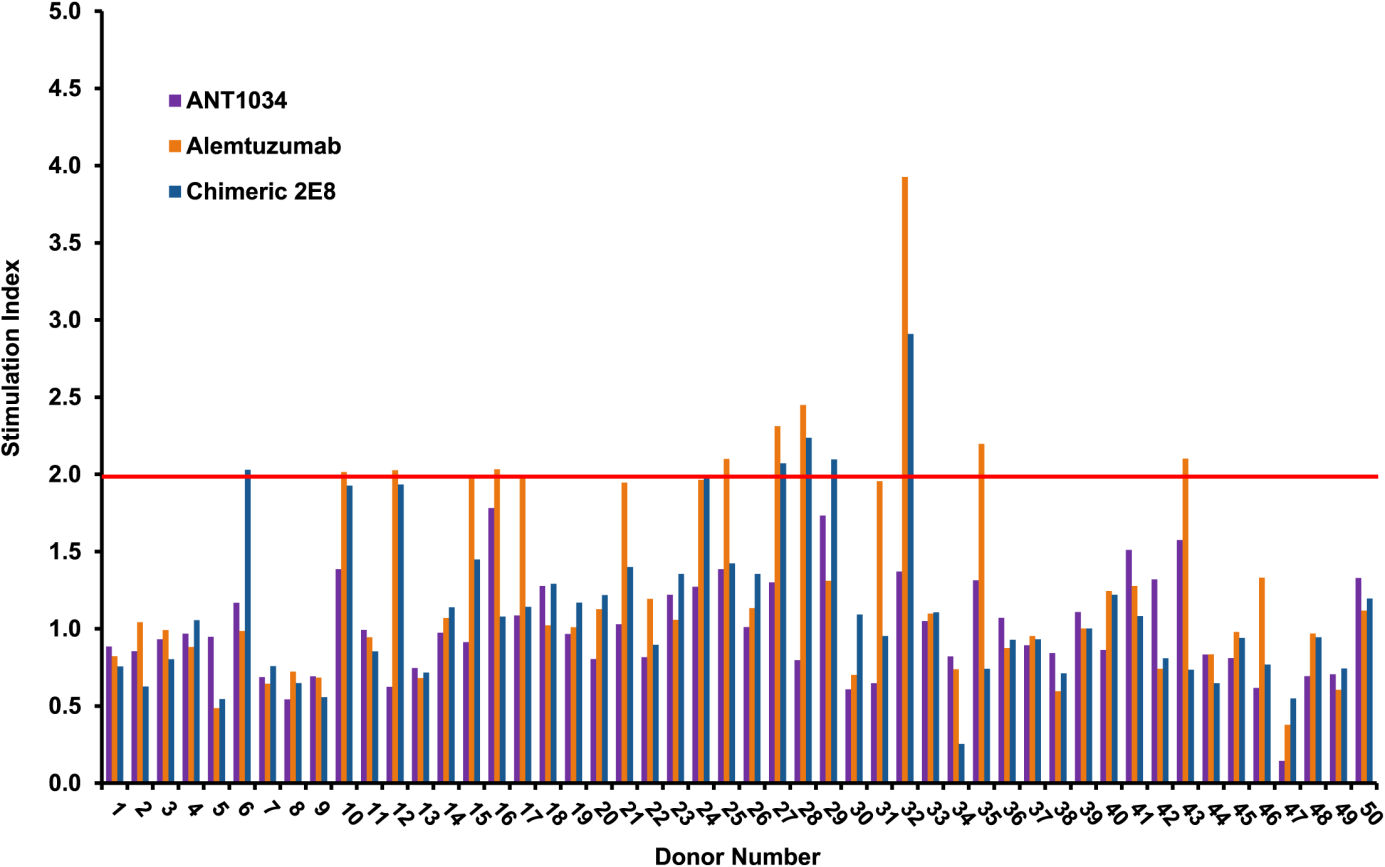
CD4+ Episcreen™ T cell proliferation responses to ANT1034, alemtuzumab and chimeric 2E8. CD4^+^ T cells were incubated with autologous mature DC loaded with the samples and assessed for proliferation after 7 days incubation. T cell responses with an SI ≥1.90 (indicated by red dotted line) that were significant (*p* <0.05) using an unpaired, two sample Student’s *t*-test were considered positive.

In order to assess the potential immunogenicity of ANT1034 in comparison to chimeric 2E8 antibody and alemtuzumab, CD4+ T cell responses were measured using *ex vivo* T cell assays. After incubation of matured DC, preloaded with test antibodies, with autologous CD4^+^ T cells for seven days, alemtuzumab and chimeric 2E8 antibody induced T cell responses in 28% and 14% of the donor cohort, respectively, whereas no responses were seen to ANT1034 (Fig 9). Chimeric 2E8, despite not being humanized, had a reduced propensity to stimulate CD4+ T cell responses compared to alemtuzumab and immunogenicity was further reduced by the rational sequence design of the ANT1034 lead variant (by avoiding CD4+ T cell epitopes).

## Discussion

Anti-CD52 targeting is a very promising therapy for B cell malignancies and autoimmune diseases including rheumatoid arthritis and multiple sclerosis; however, the current standard treatment, alemtuzumab, produces ADAs in a high frequency of patients [27][28]. In order to address this problem we have generated a novel humanized antibody, ANT1034, specific for human CD52 using Composite Human Antibody technology whereby the inclusion of CD4+ T cell epitopes in the variable domain have been avoided thus reducing its potential to be immunogenic in patients. The reference antibody was developed using a mouse immunisation regime specifically designed to focus the binding specificity on the CD52 peptide whilst boosting with CD52 expressing cells to ensure recognition of the correct conformation. This approach allowed for screening, by peptide ELISA, of large numbers of hybridomas thus increasing the likelihood of identifying an antibody with the desired properties, although a large number of peptide-positive hybridomas did not recognise CD52 on cells. The ability of ANT1034 to bind CD52 peptide sets it apart from alemtuzumab, which only weakly recognises this peptide [42]; nevertheless ANT1034 competed effectively with alemtuzumab for binding to CD52 on cells, thus indicating that the ANT1034 epitope significantly overlaps that of alemtuzumab.

In binding studies using CD52 on REH^++^ cells ANT1034 gave a substantially higher maximum signal by flow cytometry than alemtuzumab, suggesting that ANT1034 coated the cells with a higher density of antibody than alemtuzumab. This could be due either to the specific epitope recognised being more surface available for antibody binding, or to a higher overall affinity (or a combination of both). Assays for effector function demonstrated that ANT1034 was significantly more effective at killing cells by ADCC and CDC than alemtuzumab and could achieve the same level of cell killing as alemtuzumab with 100x and 10x less antibody in each assay, respectively. One possible mechanism for enhanced cell killing could be due to the increased cell surface decoration (and hence the efficiency of staining cells by flow cytometry) with ANT1034 which may result in improved ADCC responses due to the increased availability of antibody Fc [43]. Similarly, ANT1034 was able to mediate direct cell killing in the presence of a cross-linking anti-Fc F(ab’)_2_ fragment, but in the absence of both effector cells and complement, with greater efficiency than alemtuzumab. During the incubation time used in the assay, ANT1034 induced approximately twice as many cells to become necrotic as alemtuzumab, whilst the numbers of apoptotic cells was broadly similar between the antibodies. ANT1034 was investigated *in vivo* in a repeat dose CD52+ Burkitt lymphoma mouse xenograft study. ANT1034 produced superior survival rates compared to alemtuzumab even at the lowest dose administered (1 mg/Kg). In order to investigate further the enhanced activity observed for ANT1034 over alemtuzumab, the carbohydrate profile was assessed for each of the two antibodies. Carbohydrate analysis of the two antibodies revealed that they have very similar glycoprofiles (S1 Fig) which indicates that the differences in activity are not a result of differential glycosylation but are more likely to be a result of the differences between the primary amino acid sequences of the variable domains of the two antibodies and their associated antigen binding properties.

Given that the known propensity of alemtuzumab to induce dose-related infusion reactions is related to its mechanism of action [44], it may be the case that in a clinical setting the main benefit of the improved activity of ANT1034 is realised as a reduction in the dose administered. In this regard, ANT1034 was compared to alemtuzumab in a whole blood cytokine release assay and was observed to induce cytokine release (TNF-α, IL6, IL8 and IL10) at a similar level to alemtuzumab (differences between the two antibodies were not statistically significant (p < 0.05) at all the concentrations tested) (S2 Fig). Thus, despite the link in mechanism between ADCC and cytokine release, the enhanced ADCC observed with ANT1034 does not translate to a similar increase in cytokine release.

Finally, the potential immunogenicity of ANT1034 was evaluated in an *ex vivo* T cell assay where ANT1034, chimeric 2E8 and alemtuzumab-loaded and matured DC were cultured for seven days in the presence of autologous CD4+ T cells. Significant CD4+ T cell responses were observed against alemtuzumab which correlates well with the frequency of patients developing anti-therapeutic antibody responses [29]. In contrast, humanized ANT1034 failed to elicit any CD4+ T cell responses in the donor cohort thus confirming successful avoidance of T cell epitopes during the humanization design process.

Therefore we have developed a novel humanized anti-CD52 antibody, ANT1034, that has a low potential for clinical immunogenicity and improved activity which may enable reduced dosing, improved efficacy and pharmacokinetics and reduced injection site reactions. However, the data presented in this manuscript represents the first stage of a development pathway whereby the molecule will be required to progress from the relatively simple *in vitro* and *in vivo* animal studies described here to more relevant and more complex models, e.g. non-human primate studies and ultimately human clinical trials, in order to truly demonstrate its efficacy.

## Supporting Information

S1 Fig Glycoprofile analysis of alemtuzumab and ANT1034. (TIF)

S2 Fig Cytokine release comparison between alemtuzumab and ANT1034. (TIF)

## References

[1] Xia MQ, Hale G, Lifely MR, Ferguson MA, Campbell D, Packman L, et al Structure of the CAMPATH-1 antigen, a glycosylphosphatidylinositol-anchored glycoprotein which is an exceptionally good target for complement lysis. Biochem J. 1993 8 1 10.1042/bj2930633 PMC1134413 7688956

[2] Hale G. The CD52 antigen and development of the CAMPATH antibodies. Cytotherapy. Elsevier; 2001 1;3(3):137–43. 10.1080/146532401753174098 12171721

[3] Watanabe T, Masuyama J, Sohma Y, Inazawa H, Horie K, Kojima K, et al CD52 is a novel costimulatory molecule for induction of CD4+ regulatory T cells. Clin Immunol. 2006 9 10.1016/j.clim.2006.05.006 16797237

[4] Masuyama J, Yoshio T, Suzuki K, Kitagawa S, Iwamoto M, Kamimura T, et al Characterization of the 4C8 antigen involved in transendothelial migration of CD26(hi) T cells after tight adhesion to human umbilical vein endothelial cell monolayers. J Exp Med. 1999 3 15 10.1084/jem.189.6.979 PMC2193050 10075981

[5] Rowan WC, Hale G, Tite JP, Brett SJ. Cross-linking of the CAMPATH-1 antigen (CD52) triggers activation of normal human T lymphocytes. Int Immunol. 1995 1 10.1093/intimm/7.1.69 7718516

[6] Riechmann L, Clark M, Waldmann H, Winter G. Reshaping human antibodies for therapy. Nature. 1988 3 24 10.1038/332323a0 3127726

[7] Hale G. Synthetic peptide mimotope of the CAMPATH-1 (CD52) antigen, a small glycosylphosphatidylinositol-anchored glycoprotein. Immunotechnology. 1995 12 10.1016/1380-2933(95)00017-8 9373346

[8] Zent CS, Secreto CR, LaPlant BR, Bone ND, Call TG, Shanafelt TD, et al Direct and complement dependent cytotoxicity in CLL cells from patients with high-risk early-intermediate stage chronic lymphocytic leukemia (CLL) treated with alemtuzumab and rituximab. Leuk Res. 2008 12 10.1016/j.leukres.2008.05.014 PMC2588544 18584865

[9] Hu Y, Turner MJ, Shields J, Gale MS, Hutto E, Roberts BL, et al Investigation of the mechanism of action of alemtuzumab in a human CD52 transgenic mouse model. Immunology. 2009 10 10.1111/j.1365-2567.2009.03115.x PMC2767316 19740383

[10] Mone AP, Cheney C, Banks AL, Tridandapani S, Mehter N, Guster S, et al Alemtuzumab induces caspase-independent cell death in human chronic lymphocytic leukemia cells through a lipid raft-dependent mechanism. Leukemia. 2006 3 10.1038/sj.leu.2404014 16341049

[11] Stanglmaier M, Reis S, Hallek M. Rituximab and alemtuzumab induce a nonclassic, caspase-independent apoptotic pathway in B-lymphoid cell lines and in chronic lymphocytic leukemia cells. Ann Hematol. 2004 10 10.1007/s00277-004-0917-0 15309525

[12] Faulkner RD, Craddock C, Byrne JL, Mahendra P, Haynes AP, Prentice HG, et al BEAM-alemtuzumab reduced-intensity allogeneic stem cell transplantation for lymphoproliferative diseases: GVHD, toxicity, and survival in 65 patients. Blood. 2004 1 15 10.1182/blood-2003-05-1406 12969983

[13] Gupta V, Ball SE, Yi Q, Sage D, McCann SR, Lawler M, et al Favorable effect on acute and chronic graft-versus-host disease with cyclophosphamide and in vivo anti-CD52 monoclonal antibodies for marrow transplantation from HLA-identical sibling donors for acquired aplastic anemia. Biol Blood Marrow Transplant. 2004 12 10.1016/j.bbmt.2004.09.001 15570255

[14] Keating MJ, Flinn I, Jain V, Binet J-L, Hillmen P, Byrd J, et al Therapeutic role of alemtuzumab (Campath-1H) in patients who have failed fludarabine: results of a large international study. Blood. 2002 5 15 [cited 2015 Jan 30];99(10):3554–61. Available from: http://www.ncbi.nlm.nih.gov/pubmed/11986207 11986207 10.1182/blood.v99.10.3554

[15] Brett SJ, Baxter G, Cooper H, Rowan W, Regan T, Tite J, et al Emergence of CD52-, glycosylphosphatidylinositol-anchor-deficient lymphocytes in rheumatoid arthritis patients following Campath-1H treatment. Int Immunol. 1996 3 10.1093/intimm/8.3.325 8671618

[16] Isaacs JD, Watts RA, Hazleman BL, Hale G, Keogan MT, Cobbold SP, et al Humanised monoclonal antibody therapy for rheumatoid arthritis. Lancet. 1992 9 26 10.1016/0140-6736(92)92294-p 1356177

[17] Matteson EL, Yocum DE, St Clair EW, Achkar AA, Thakor MS, Jacobs MR, et al Treatment of active refractory rheumatoid arthritis with humanized monoclonal antibody CAMPATH-1H administered by daily subcutaneous injection. Arthritis Rheum. 1995 9 10.1002/art.1780380903 7575711

[18] Lundin J, Osterborg A, Brittinger G, Crowther D, Dombret H, Engert A, et al CAMPATH-1H monoclonal antibody in therapy for previously treated low-grade non-Hodgkin’s lymphomas: a phase II multicenter study. European Study Group of CAMPATH-1H Treatment in Low-Grade Non-Hodgkin's Lymphoma. J Clin Oncol. 1998 10 10.1200/JCO.1998.16.10.3257 9779699

[19] Osterborg A, Werner A, Halapi E, Lundin J, Harmenberg U, Wigzell H, et al Clonal CD8+ and CD52- T cells are induced in responding B cell lymphoma patients treated with Campath-1H (anti-CD52). Eur J Haematol. 1997 1 10.1111/j.1600-0609.1997.tb01403.x 9020367

[20] Ginaldi L, De Martinis M, Matutes E, Farahat N, Morilla R, Dyer MJ, et al Levels of expression of CD52 in normal and leukemic B and T cells: correlation with in vivo therapeutic responses to Campath-1H. Leuk Res. 1998 3 10.1016/s0145-2126(97)00158-6 9593475

[21] Pawson R, Dyer MJ, Barge R, Matutes E, Thornton PD, Emmett E, et al Treatment of T-cell prolymphocytic leukemia with human CD52 antibody. J Clin Oncol. 1997 7 10.1200/JCO.1997.15.7.2667 9215839

[22] Garnock-Jones KP. Alemtuzumab: a review of its use in patients with relapsing multiple sclerosis. Drugs. 2014 3 10.1007/s40265-014-0195-7 24604792

[23] Jones JL, Coles AJ. Mode of action and clinical studies with alemtuzumab. Exp Neurol. 2014 12 10.1016/j.expneurol.2014.04.018 24792641

[24] Alinari L, Lapalombella R, Andritsos L, Baiocchi RA, Lin TS, Byrd JC. Alemtuzumab (Campath-1H) in the treatment of chronic lymphocytic leukemia. Oncogene. 2007 5 28 10.1038/sj.onc.1210380 17530018

[25] Dearden CE, Matutes E. Alemtuzumab in T-cell lymphoproliferative disorders. Best Pract Res Clin Haematol. 2006 1 10.1016/j.beha.2006.05.005 16997184

[26] Enblad G, Hagberg H, Erlanson M, Lundin J, MacDonald AP, Repp R, et al A pilot study of alemtuzumab (anti-CD52 monoclonal antibody) therapy for patients with relapsed or chemotherapy-refractory peripheral T-cell lymphomas. Blood. 2004 4 15 10.1182/blood-2003-10-3389 15070664

[27] Weinblatt ME, Maddison PJ, Bulpitt KJ, Hazleman BL, Urowitz MB, Sturrock RD, et al CAMPATH-1H, a humanized monoclonal antibody, in refractory rheumatoid arthritis. An intravenous dose-escalation study. Arthritis Rheum. 1995 11 10.1002/art.1780381110 7488279

[28] Somerfield J, Hill-Cawthorne GA, Lin A, Zandi MS, McCarthy C, Jones JL, et al A novel strategy to reduce the immunogenicity of biological therapies. J Immunol. American Association of Immunologists; 2010 7 1 10.4049/jimmunol.1000422 20519651

[29] Baker MP, Jones TD. Identification and removal of immunogenicity in therapeutic proteins. Curr Opin Drug Discov Devel. 2007 3;10(2):219–27. 17436557

[30] Bryson CJ, Jones TD, Baker MP. Prediction of immunogenicity of therapeutic proteins: validity of computational tools. BioDrugs. 2010 3 1 10.2165/11318560-000000000-00000 20055528

[31] Almagro JC, Fransson J. Humanization of antibodies. Int Immunol. 2012 7;24(7):1620–33.

[32] Jones TD, Crompton LJ, Carr FJ, Baker MP. Deimmunization of monoclonal antibodies. Methods Mol Biol. 2009 1;525:405–23, xiv. 10.1007/978-1-59745-554-1_21 19252848

[33] Perry LCA, Jones TD, Baker MP. New approaches to prediction of immune responses to therapeutic proteins during preclinical development. Drugs R D. 2008 1 10.2165/0126839-200809060-00004 18989990

[34] Baum C, Forster P, Hegewisch-Becker S, Harbers K. An optimized electroporation protocol applicable to a wide range of cell lines. Biotechniques. 1994 12;17(6):1058–62. 7873174

[35] Jones S, Bendig MM. Rapid PCR-cloning of full-length mouse immunoglobulin variable regions. Bio/technology. 1991;9(January):88–9. 1367218 10.1038/nbt0191-88

[36] Jordan M, Köhne C, Wurm FM. Calcium-phosphate mediated DNA transfer into HEK-293 cells in suspension: control of physicochemical parameters allows transfection in stirred media. Transfection and protein expression in mammalian cells. Cytotechnology. 1998 1 10.1023/A:1007917318181 PMC3449510 22359005

[37] Guex N, Peitsch MC. SWISS-MODEL and the Swiss-PdbViewer: an environment for comparative protein modeling. Electrophoresis. 1997 12;18(15):2714–23. 9504803 10.1002/elps.1150181505

[38] Golay J, Manganini M, Facchinetti V, Gramigna R, Broady R, Borleri G, et al Rituximab-mediated antibody-dependent cellular cytotoxicity against neoplastic B cells is stimulated strongly by interleukin-2. Haematologica. 2003 9 12969808

[39] Lapalombella R, Zhao X, Triantafillou G, Yu B, Jin Y, Lozanski G, et al A novel Raji-Burkitt’s lymphoma model for preclinical and mechanistic evaluation of CD52-targeted immunotherapeutic agents. Clin Cancer Res. 2008;14(2):569–78. 10.1158/1078-0432.CCR-07-1006 18223233

[40] Shi B, Ma L, He X, Wang X, Wang P, Zhou L, et al Comparative analysis of human and mouse immunoglobulin variable heavy regions from IMGT/LIGM-DB with IMGT/HighV-QUEST. Theor Biol Med Model. 2014 1 10.1186/1742-4682-11-30 PMC4085081 24992938

[41] Kabat EA. Sequences of proteins of immunological interest. NIH Publication. 1991

[42] Hale G. Synthetic peptide mimotope of the CAMPATH-1 (CD52) antigen, a small glycosylphosphatidylinositol-anchored glycoprotein. Immunotechnology. 1995 12;1(3–4):175–87. 9373346 10.1016/1380-2933(95)00017-8

[43] Tang Y, Lou J, Alpaugh RK, Robinson MK, Marks JD, Weiner LM. Regulation of Antibody-Dependent Cellular Cytotoxicity by IgG Intrinsic and Apparent Affinity for Target Antigen. J Immunol. American Association of Immunologists; 2007 8;179(5):2815–23. 17709495 10.4049/jimmunol.179.5.2815

[44] Hansel TT, Kropshofer H, Singer T, Mitchell JA, George AJT. The safety and side effects of monoclonal antibodies. Nat Rev Drug Discov. Nature Publishing Group; 2010 4;9(4):325–38. 10.1038/nrd3003 20305665

