# Supplementary figures and images for: Characterisation of a Novel Anti-CD52 Antibody with Improved Efficacy and Reduced Immunogenicity
Source: PLoS One. 2015 Sep 15;10(9):e0138123. doi: 10.1371/journal.pone.0138123 (PMC4570798; doi:10.1371/journal.pone.0138123)

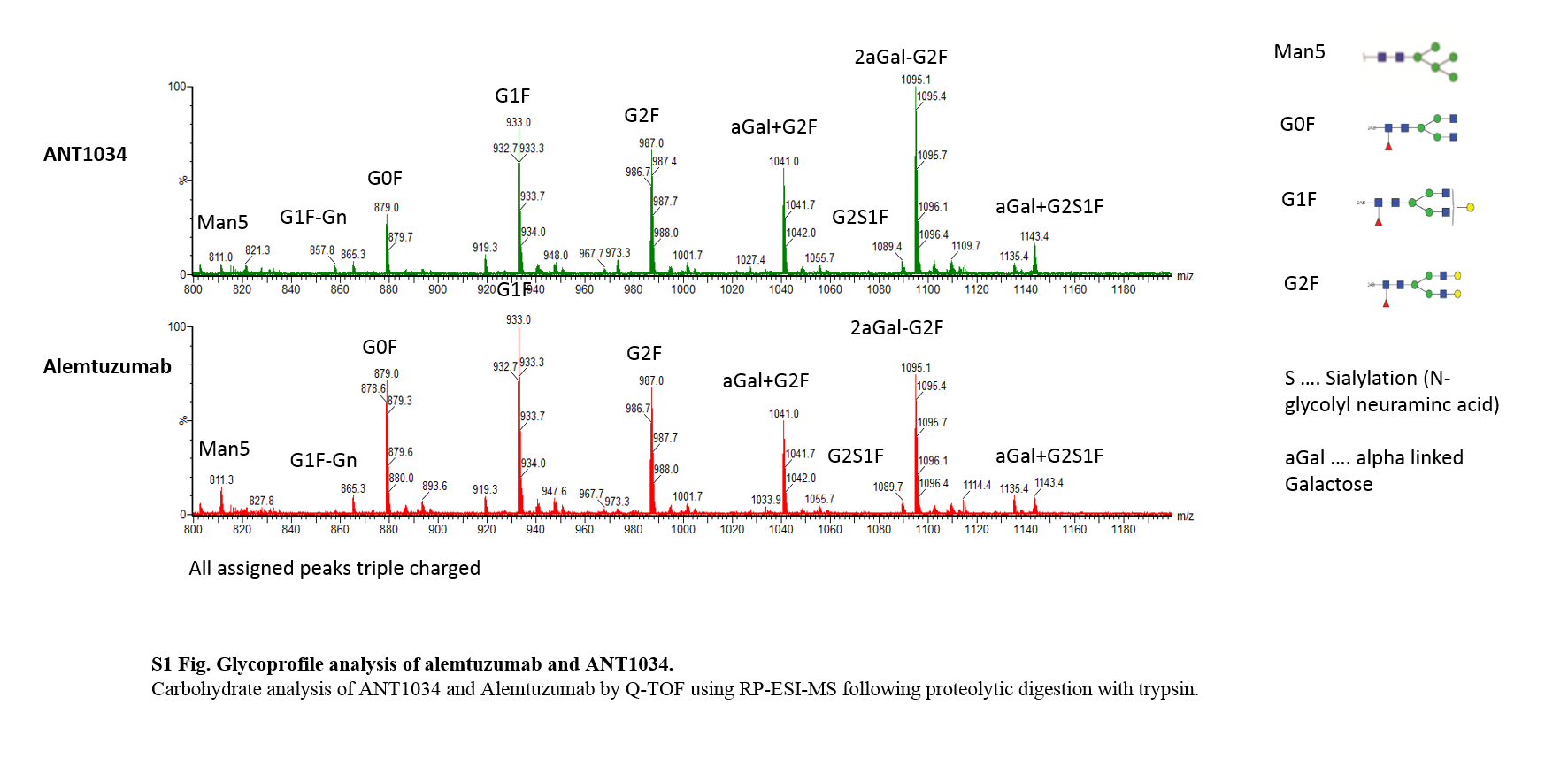

Supplement: S1 Fig — (TIF) [file pone.0138123.s001.tif]
